# JN.1 as a new variant of COVID-19 – editorial

**DOI:** 10.1097/MS9.0000000000001876

**Published:** 2024-02-28

**Authors:** Mizbahul Karim Hemo, Md. Aminul Islam

**Affiliations:** a Advance Molecular Genetics Lab, Department of Microbiology, Primeasia University, Star Tower & HBR, Dhaka; b COVID-19 Diagnostic Lab, Department of Microbiology, Noakhali Science and Technology University, Noakhali; c Advanced Molecular Lab, Department of Microbiology, President Abdul Hamid Medical College, Karimganj, Kishoreganj, Bangladesh

## Introduction

The global landscape of the ongoing coronavirus disease 2019 (COVID-19) pandemic continues to evolve, marked by the emergence of new severe acute respiratory syndrome coronavirus 2 (SARS-CoV-2) variants that demand heightened vigilance and monitoring. In a significant development, the World Health Organization (WHO) has recently elevated the status of a novel strain, JN.1, designating it as a ‘variant of interest,’ underlining its potential significance in the ongoing battle against the virus. Concurrently, the Centers for Disease Control and Prevention (CDC) in the United States (U.S.) employs a comprehensive and diverse surveillance approach to monitor the emergence and spread of such variants, exemplifying the collaborative effort on a global scale^1^.

Originating in Denmark in late July, JN.1 has swiftly traversed borders and been identified in various countries, including Canada, Israel, Portugal, South Africa, Sweden, the United Kingdom (U.K.), and the U.S. The variant is distinguished by a multitude of mutations within the spike gene, adding a layer of complexity to our understanding of the virus’s behavior. This discovery has prompted an intensified surveillance effort, encompassing genomic analysis, wastewater monitoring, traveler-based tracking, and digital public health surveillance, synergizing with established public health systems^2^. JN.1, a lineage descended from BA.2.86, has been the subject of extensive analysis since the 25 August 2023 sample collection date. Notably, a distinctive L455S mutation in the spike protein sets JN.1 apart from its parent lineage, BA.2.86. The heightened concern surrounding the BA.2.86 strain, also known as ‘Pirola,’ escalated following the detection of an outbreak in a care facility in the U.K., leading the government to declare its probable spread within the community^3^.

As the global community assesses the potential public health impact of JN.1, current evaluations suggest a low level of additional risk on a global scale. However, given the approaching winter season, countries are advised to remain vigilant. The co-circulation of SARS-CoV-2 and other pathogens may pose challenges, potentially exacerbating the respiratory disease burden. This article delves into the intricate details of JN.1, shedding light on its characteristics, spread, and the collective global effort to understand and mitigate the risks associated with this new variant^4^.

## Global prevalence

As of 16 December 2023, the world is witnessing a transformative chapter in the COVID-19 saga, marked by the ascent of the JN.1 variant. Data compiled from 41 countries and submitted to the Global Initiative on Sharing All Influenza Data (GISAID) underscores the magnitude of this shift, with 7344 JN.1 sequences representing a formidable 27.1% of globally available sequences during epidemiological week 48 (27 November to 3 December 2023). This unprecedented prevalence vividly shows the variant’s expansive reach and impact across continents^5^.

Leading the charge in JN.1 reporting are notable nations, with France at the forefront, contributing a substantial 20.1% (1552 sequences) of the global dataset. The U.S. closely follows with 14.2% (1072 sequences), underscoring the variant’s rapid establishment within the nation. Singapore, Canada, the U.K., and Sweden contribute 12.4%, 6.8%, 5.6%, and 5.0%, respectively, highlighting the variant’s global footprint.

When the CDC data is examined closely, an unsettling story emerges. With a startling 15–29% of new infections, JN.1 has quickly become the COVID-19 strain with the fastest rate of growth in the nation. With one-third of all cases documented, it has already established its dominance in the Northeast. A 10.5% spike in mortality and an 8.7% increase in hospital admissions were reported in the U.S. during the week ending 2 September 2023, which highlights the severity of the situation given the impending mid-September launch of the 2023–2024 COVID-19 vaccine.

While these statistics may instill concern, the CDC offers a glimmer of hope, suggesting that early analysis indicates existing antibodies may be effective against the new BA.2.86 variant, presenting a potential avenue for combating the impact of JN.1. This article delves into the unfolding global prevalence scenario, providing insights into the variant’s widespread distribution, its impact on nations at the forefront, and the challenges and opportunities it poses for the ongoing global battle against the evolving COVID-19 landscape^6^.

## Detection surveillance

Early identification of novel SARS-CoV-2 variants is essential for risk assessment, public health outreach, and communication. The CDC identified and tracked the JN.1 variant worldwide using a variety of surveillance techniques, such as genomic, wastewater, traveler-based, and digital public health surveillance^7^.

1. National SARS-CoV-2 Genomic Surveillance: for the National SARS-CoV-2 genomic surveillance, three different sequence sources were used:
  - These sources were combined and modeled in order to produce weighted estimates of variant proportions that were systematically estimated over 2-week intervals.
  - Globally sourced SARS-CoV-2 sequences were submitted to the Global Initiative on Sharing All Influenza Data (GISAID) and the National Centre for Biotechnology’s Information Sequence Read Archive (NCBI SRA).
  - A detailed grasp of the dynamics and prevalence of variants throughout time was possible thanks to the weighted estimations.
2. Traveler-based Genomic Surveillance (TGS):
  - TGS was used as a unique monitoring strategy, concentrating on genetic information from people who had traveled.
  - Travelers’ genetic data provide insights into the possibility of SARS-CoV-2 genotypes spreading across international borders.
  - This system has aided a thorough understanding of variation in mobility and distribution across geographic borders.
3. National Wastewater Surveillance System (NWSS):
  - NWSS played a key role in wastewater analysis-based SARS-CoV-2 prevalence monitoring.
  - The identification of variation hotspots was aided by the community-level perspective that wastewater surveillance gave.
  - The information obtained from the NWSS provided light on the wider spread of SARS-CoV-2 variations in different populations.
4. Digital Public Health Surveillance:
  - Digital public health surveillance is a multi-faceted approach encompassing various data sources.
  - Monitoring of global public genomic data repositories, such as NCBI and GISAID, allowed for real-time tracking of genomic data trends.
  - Additional sources, including news media, social media, and global event-based reports from public health partners, were integrated into the surveillance system.
  - This comprehensive approach facilitated the rapid identification of emerging trends and potential public health threats associated with SARS-CoV-2 variants.

## Clinical manifestation

The WHO and the CDC do not routinely gather information on how COVID symptoms are changing over time, making it difficult to determine whether infections are manifesting differently. But medical professionals said they have not seen anything unusual. According to Dr Molly Fleece, a hospital epidemiologist at the University of Alabama at Birmingham Medicine, ‘the symptoms of JN.1 seem to be very similar, if not the same, as others.’ A significant number of people who have recently been diagnosed with COVID have indicated that their initial symptoms were sore throats, which were frequently followed by congestion. Symptoms like a dry cough and a diminished sense of smell or taste, which were once prevalent, are now less frequently reported, say medical professionals. However, severe cases are still marked by symptoms such as difficulty breathing, chest discomfort, or anemia, which manifest as a shortage of oxygen in the skin, lips, or nail beds. However, compared to when the pandemic first began, symptoms of COVID-19 are generally less severe^8^.

## Integration and analysis

1. Data from all four surveillance systems were collated and analyzed collectively to provide a holistic perspective on the prevalence, movement, and characteristics of SARS-CoV-2 variants.
2. The integration of multiple surveillance approaches allowed for cross-validation and a more robust understanding of the evolving dynamics of the virus.

## Limitations

While these surveillance systems provide valuable insights, it is important to acknowledge inherent limitations, such as potential biases in sampling and reporting, which may influence the accuracy of the results. Continuous refinement and validation of the surveillance methodologies are imperative for ensuring the reliability of the findings.

## Current vaccines

As of December 2023, the U.S. CDC assures that the newly identified JN.1 variant, along with its precursor BA.2.86, exhibits only a single change in the spike protein – a critical component targeted by existing COVID-19 vaccines. Given this minimal difference, current vaccines are expected to remain effective against both variants.

The CDC reports that initial scientific data on the updated 2023–2024 COVID-19 vaccines indicate their efficacy in blocking the BA.2.86 variant. The CDC anticipates similar effectiveness against JN.1, reinforcing confidence in the adaptability of existing vaccines to evolving viral strains.

The WHO emphasizes that current vaccines continue to protect against severe disease and death caused by JN.1 and different circulating SARS-CoV-2 variants. The WHO urges individuals, particularly those at high risk, to stay up to date with their vaccinations. Despite this reassurance, the WHO warns that respiratory diseases beyond COVID-19 are on the rise, including flu, respiratory syncytial virus (RSV), and common childhood pneumonia. In light of these concerns, the WHO advocates for the continuation of preventive measures, such as wearing masks, regular handwashing, covering coughs and sneezes, and staying home when sick^9^.

Epidemiologist Katelyn Jetelina highlights the rapid spread of the JN.1 variant, noting its exponential increase in public wastewater indications. Jetelina suggests that the impact on hospitalizations is a key question, particularly in regions with low vaccination rates. Notably, the U.K. and Singapore, with high vaccination rates, have observed a significant rise in hospitalizations following the dominance of JN.1. Jetelina acknowledges the challenging timing of the variant’s emergence amid expanding social networks during the holiday season but expresses optimism that the situation, though less than optimal, may not escalate to the extent witnessed with previous variants. This nuanced perspective encourages caution without necessitating a cancellation of holiday plans^10^.

## Conclusion

The JN.1. variation is the subject of ongoing research into its features and possible consequences. Variant JN.1 poses significant challenges to global COVID-19 efforts due to its unique mutations and rapid spread, despite existing antibodies providing some protection, while current vaccines show significant promise. Genomic analysis, immune system dynamics, wastewater monitoring, and determining its impact on susceptible populations are all components of this comprehensive scientific investigation. The knowledge gained will help improve public health strategies and safeguard public health by staying at the forefront of research and surveillance. This continually developing viral adversary requires a comprehensive and proactive response that integrates scientific insights. By leading research and surveillance, we can protect public health and respond globally to the JN.1 variant’s complex effects.

## Ethical approval

Ethics approval was not required for this editorial.

## Consent

Informed consent was not required for this editorial.

## Sources of funding

Not applicable.

## Author contribution

M.K.H. and M.A.I.: conceptualization, data curation, investigation, writing – original draft, writing – review and editing, validation, and reviewing. Both authors carefully checked and approved the version.

## Conflicts of interest disclosure

The author declares no conflicts of interest.

## Research registration unique identifying number (UIN)

1. Name of the registry: not applicable.
2. Unique identifying number or registration ID: not applicable.
3. Hyperlink to your specific registration (must be publicly accessible and will be checked): not applicable.

## Guarantor

Mizbahul Karim Hemo (MSc in Microbiology), Lab Instructor (Research Officer), Department of Microbiology, Primeasia University, Star Tower & HBR, 12 Kemal Ataturk Ave, Dhaka 1213, Orchid ID: https://orcid.org/0000-0003-4818-8260, E-mail: bdmizba@gmail.com, Bdmizba@primeasia.edu.bd; Tel/WhatsApp: +880 1779123647, https://www.webofscience.com/wos/author/record/HGU-5145-2022, SciProfiles: 2890043, Google Scholar: https://scholar.google.com/citations?user=0h-6IuwAAAAJ&hl=en, Web address: https://microbiology.primeasia.edu.bd/dept-faculty-members1.

## Data availability statement

Not applicable.

## Provenance and peer review

Not applicable.
